# Higher risk of dementia in English older individuals who are overweight or obese

**DOI:** 10.1093/ije/dyaa099

**Published:** 2020-06-23

**Authors:** Yixuan Ma, Olesya Ajnakina, Andrew Steptoe, Dorina Cadar

**Affiliations:** d1 Department of Behavioural Science and Health, University College London, London, UK; d2 Department of Biostatistics & Health Informatics, Institute of Psychiatry, Psychology and Neuroscience, King’s College London, London, UK

**Keywords:** Obesity, body mass index, dementia, longitudinal study

## Abstract

**Background:**

Several risk factors contribute to dementia, but the role of obesity remains unclear. This study investigated whether increased body weight or central obesity were associated with a higher risk of developing dementia in a representative sample of older English adults.

**Methods:**

We studied 6582 participants from the English Longitudinal Study of Ageing (ELSA) who were aged ≥50 years and were dementia-free at baseline, that being either wave 1 (2002–2003) for study members who started at wave 1, or at either wave 2 (2004–2005) or 4 (2008–2009) for those who began the study as refreshment samples. Body mass index (BMI) was measured at baseline and categorized into normal weight (18.5–24.9 kg/m^2^), overweight (25–29.9 kg/m^2^) and obese (≥30 kg/m^2^). Central obesity was defined as a waist circumference (WC) >88 cm for women and >102 cm for men. Cumulative incidence of dementia was ascertained based on physician-diagnosed dementia, an overall score >3.38 on the Informant Questionnaire on Cognitive Decline in the Elderly (IQCODE) and Hospital Episodes Statistics (HES) data at every ELSA wave from baseline until wave 8 (2016–2017). Cox proportional hazards models were used to assess the association between baseline BMI levels or abdominal obesity in relation to dementia incidence during the mean follow-up period of 11 years.

**Results:**

From the overall sample, 6.9% (*n* = 453) of participants developed dementia during the follow-up period of maximum 15 years (2002–2017). Compared with participants with normal weight, those who were obese at baseline had an elevated risk of dementia incidence [hazard ratio (HR) = 1.34, 95% confidence interval (CI) 1.07–1.61] independent of sex, baseline age, apolipoprotein E-ε4 (APOE-ε4), education, physical activity, smoking and marital status. The relationship was slightly accentuated after additionally controlling for hypertension and diabetes (HR = 1.31, 95% CI 1.03–1.59). Women with central obesity had a 39% greater risk of dementia compared with non-central obese women (HR = 1.39, 95% CI 1.12–1.66). When compared with a normal BMI and WC group, the obese and high WC group had 28% (HR = 1.28, 95% CI 1.03–1.53) higher risk of dementia.

**Conclusions:**

Our results suggest that having an increased body weight or abdominal obesity are associated with increased dementia incidence. These findings have significant implications for dementia prevention and overall public health.

**Key Messages**

- In a representative sample of English older adults, 74% of the participants who developed dementia were overweight or obese at baseline, in comparison with 72% of those who were not diagnosed with dementia by the end of the study period.
- Obesity and larger waist circumference were associated with increased dementia incidence. These findings have significant implications for dementia prevention and overall public health associated with a higher dementia risk across a decade follow-up period, independent of demographics, lifestyle behaviours, apolipoprotein E-ε4, hypertension and diabetes
- From the various modifiable risk factors, obesity could represent a target for intervention, and these findings have significant implications for public health and dementia prevention.

## Introduction

Dementia is a substantial public health burden^1^ ^2^ and has become one of the leading causes of mortality in England, accounting for more than one in eight of all deaths (12.7%) in 2017.^3^ Therefore, the identification of the modifiable risk factors for dementia onset has become a public health priority.^4^ Obesity is one of the modifiable risk factors associated with cardiovascular disease,^5^ stroke^6^ and dementia.^7–14^ In 2016, 39% of adults were estimated to be overweight,^15^ while the global prevalence of dementia had almost tripled from 1975 to 2016.^16^ ^17^ Biologically, excess body fat is linked with a change in energy metabolism,^11^ the accumulation of brain lesions and detectable brain volume deficits,^18^ the latter being an early marker of neurodegeneration.^19^

Nonetheless, the evidence for the link between higher body mass index (BMI) and dementia onset is not entirely clear. A retrospective study of 2 million electronic records of patients from the UK Clinical Practice Research Datalink (CPRD) showed that mid-life obesity was associated with substantially reduced dementia risk.^20^ However, some studies showed a null association,^21^ ^22^ even when a Mendelian randomization approach was considered,^23^ ^24^ whereas others suggested that obesity represents a more significant risk factor for women than men.^25^ In terms of temporality across the life course, some studies provide support for the link between higher BMI and an increased risk of dementia onset,^22^ ^25^ although higher BMI in older age (≥70 years) has been shown to be protective against dementia risk.^22^ These findings raise uncertainty in understanding the underlying link between body fat and dementia aetiology. In addition, there are also claims that obesity may be protective and associated with higher survival in elderly individuals, representing evidence for reverse causality, also known as the ‘obesity paradox’,^26^ which further compounds our current understanding of the relationship between obesity and dementia risk in older adults. Furthermore, some studies suggest that waist circumference (WC), an indicator of abdominal obesity, might be a more sensitive adiposity marker than BMI,^27^ and provided evidence to support the link between high adiposity and a greater dementia risk,^13^ ^28^ with a stronger relationship particularly among older adults with larger WC.^29^ However, two longitudinal studies found no association between late-life WC and dementia risk.^12^ ^30^

Therefore, the present study aimed to investigate the relationship between obesity and dementia risk in a representative population sample of English adults, aged ≥50 years (mean age of 63 years at baseline) who have been followed-up for up to 15 years. It was hypothesized that individuals with higher BMI levels and increased WC would be associated with an increased dementia incidence during the follow-up period, compared with those with lower levels.

## Methods

### Sample

**Figure 1.**
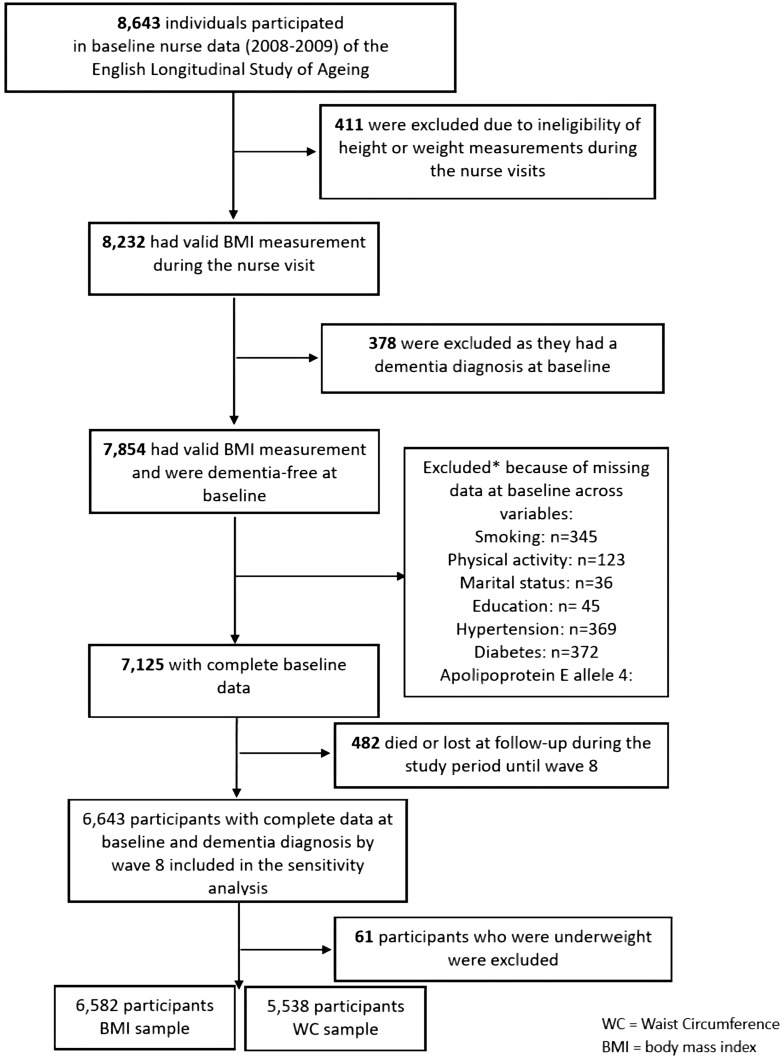
The flow of participants throughout the study.

The target population, the English Longitudinal Study of Ageing (ELSA) consisted of initially 11 391 nationally representative men and women (≥50 years of age), living in the community in England in 2002–2003.^31^ Participants were interviewed biannually, whereas the nurse interviews were conducted every 4 years. The baseline wave was either wave 1 (2002–2003) for core members who started the study at wave 1, or wave 2 (2004–2005) or 4 (2008–2009) for core members who joined the study as refreshment samples at later waves. The latest wave available at the time of these analyses was wave 8 (2016–2017), and therefore the analyses used in this study used data spanning across up to 15 years follow-up (2002–2017). For the BMI analysis, the sample was comprised of 6582 participants (62.6 ± 9.0 years; 46.0% men), whereas for the WC analyses, the sample was comprised of 5538 participants (63.5 ± 9.3 years; 45.4% men) who were free from dementia at their baseline assessment and had complete cases on all variables of interest (see flow chart, Figure 1**)**. Ethical approval was obtained by the London Multi-Centre Research Ethics Committee (MREC/01/2/91) and all ELSA participants provided informed verbal consent. The ELSA data are available in a public, open-access repository (the UK Data Archive) which is freely available and can be accessed at https://discover.ukdataservice.ac.uk

### Dementia and survival age

Dementia assessment was determined using a triangulation method with three sources of information. Firstly, doctors-diagnosed dementia or Alzheimer’s disease reported between waves 5 and 8 by the participants who were capable of participating personally in the study. Secondly, the adapted short-form Informant Questionnaire on Cognitive Decline in the Elderly (IQCODE) questionnaire, which contains 16 items, used as the second criterion, has been completed by an informant (family member or long-term caregiver) to compare the present functional and cognitive performance with the prior performance during the past 2 years. On a 5-point scale, each item is scored from 1 = much improved to 5 = much worse. A threshold of 3.38 or more on the IQCODE was used to define dementia with high-sensitivity (0.82) and specificity (0.84).^32^ Third, records from the Hospital Episode Statistics were used to identify dementia patients, even if their primary cause of hospitalization was not dementia. The survival time was computed as the time elapsed from the date of baseline interview to the first of either date of dementia diagnosis, death or the latest interview before dropout or the most recent interview date available at the time of the current analyses, which corresponded to wave 8 (2016–2017).

### Anthropometric measurements

All anthropometric measures were taken by trained nurses. Weight was measured using Tanita electronic scales to measure body weight without shoes and in light clothing. Height was determined by Stadiometer using the Frankfort plane at ground level. BMI was calculated using standard formulae (weight in kilograms/height in square meters) and categorized into the following categories: underweight (BMI < 18.5 kg/m^2^), normal (18.5–24.9 kg/m^2^), overweight (25–29.9 kg/m^2^) and obese (≥30 kg/m^2^), according to the standard criteria of the World Health Organization.^33^ Due to the limited number of participants being classified as underweight in this study (*n* = 61), we excluded the underweight group from the main analyses and presented them only in a sensitivity analysis. WC was measured twice using the midpoint between the iliac crest and lower rib. The mean of the first two valid measurements was used given the difference was ≤3 cm. WC was recorded to the nearest even millimetre. Abdominal obesity was defined as WC > 88 cm for women and >102 cm for men.^34^

### Covariates

The following baseline covariates were considered: age, sex, apolipoprotein E ε4 allele (APOE-ε4) status (yes/no), education (no qualifications; A-level; degree or above), marital status (married/unmarried), current smoking (yes/no), physical activities (yes/no), hypertension (>140/90 mmHg) and diabetes (yes/no). Education and marital status were considered as confounders since evidence suggests that individuals with more years of formal educational attainment are estimated to have a lower risk of dementia.^35^ Finally, a non-modifiable risk factor, such as APOE-ε4, was included, since it is an established genetic risk factor associated with dementia.^36^ APOE-ε4 carrier status was coded as 0 for absent and 1 for present.

### Statistical analysis

Cox proportional hazards regressions were carried out to evaluate the relationship between BMI or WC levels at baseline and dementia incidence between wave 5 (2010–2011) and wave 8 (2016–2017). We also evaluated the joint association of BMI with dementia risk within WC strata (normal BMI + WC, normal BMI + high WC, obese + normal WC and obese + high WC). Four models were fitted to these analyses in a stepwise fashion to understand the role of covariates in this association. The first model was only adjusted for baseline age, sex and APOE-ε4 (Model 1). The second model was further adjusted for baseline education and marital status (Model 2). The third model additionally included vascular risk factors—smoking and physical activities (Model 3), whereas the final model was additionally adjusted for diabetes and hypertension (Model 4). To investigate the putative multiplicative interactions between obesity and APOE-ε4 status, an interaction term was included in our analyses. In addition, interactions between sex, hypertension and diabetes in relation to BMI and WC were tested. The Akaike information criterion (AIC) was used to select the model with the best model fit.^37^ The inflation factor was <1.93, suggesting that there is no evidence of multicollinearity. To test the proportional hazards assumptions of the Cox models, the scaled Schoenfeld residual test was computed, and all models met the proportional hazards assumptions (all *P* > 0.05). We used the derived baseline cross-sectional survey weights from ELSA in all analyses to ensure the sample is representative of the English population. Two-sided *P* values are presented for all the analyses. All statistical analyses were conducted using R Studio Version 3.5.2.^38^ ELSA data are freely available to any researcher registered with the UK Data Service (https://www.ukdataservice.ac.uk).

### Sensitivity analysis

Three sensitivity analyses were additionally conducted. In the first sensitivity analysis, we used BMI recoded into four groups (normal, underweight, overweight and obese) to explore the full breakdown of these BMI categories, despite that the underweight group was small. Second and third sensitivity analysis repeated the two main analyses in an imputed dataset using the missForest method of multiple imputations.^39^ Results from sensitivity analyses are available as Supplementary data at *IJE* online.

## Results

### Sample characteristics

The follow-up period of this study was up to 15 years [range = 0.08–15 years; mean = 11.4 years, standard deviation (SD) = 3.3]. Table 1 presents the baseline characteristics of participants within the first analytical sample using BMI and Table 2 shows the baseline characteristics of participants captured within the second analytical sample using WC split into dementia-diagnosed and dementia-free groups. Of the 6582 participants free of dementia at baseline for the first analytical sample, 453 (6.9%) were subsequently diagnosed with dementia, accounting for 75 220 person-years. Participants who developed dementia during follow-up were older at their baseline assessment (mean = 71.8 years, SD = 7.8) compared with dementia-free counterparts (mean = 61.9 years, SD = 8.7), *t* = −25.7, degrees of freedom = 538.1, *P* < 0.001. Of the 5538 participants in the second analytical sample, 432 (6.9%) were subsequently diagnosed with dementia. Participants who developed dementia during follow-up were older at their baseline assessment (mean = 72.2 years, SD = 7.9) compared with dementia-free counterparts (mean = 62.7 years, SD = 9.0). Individuals with dementia were more likely to be APOE-ε4 carriers, unmarried, less likely to have qualifications, and to have diabetes and hypertension and lead a sedentary life (i.e. no physical activities) compared with participants who did not develop dementia during the follow-up period (all *P* < 0.001). However, these groups did not differ substantially in terms of WC, BMI levels, gender or smoking.

**Table 1.** Baseline characteristics of the BMI analytical sample (*n* = 6582) by dementia status

	Dementia (*n* = 453)	No dementia (*n* = 6129)	*P*‐Value
BMI (categories) (kg/m^2^), *n* (%)			0.657^a^
Normal (18.5–24.9)	116 (25.6)	1691 (27.6)	
Overweight (25.0–29.9)	203 (44.8)	2683 (43.8)	
Obese (≥30.0)	134 (29.6)	1755 (28.6)	
Age (years), mean (SD)	71.8 (7.8)	61.9 (8.7)	<0.001^b^
Men, *n* (%)	204 (45.0)	2826 (46.1)	0.693^a^
APOE ε4 carrier, *n* (%)	183 (40.4)	1474 (24.0)	<0.001^a^
Education, *n* (%)			<0.001^a^
No qualifications	222 (49.0)	1971 (32.2)	
A-level	156 (34.4)	2370 (38.7)	
Higher	75 (16.6)	1788 (29.2)	
Unmarried, *n* (%)	203 (44.8)	1842 (30.1)	<0.001^a^
No physical activity, *n* (%)	32 (7.1)	179 (2.9)	<0.001^a^
Current smokers, *n* (%)	59 (13.0)	887 (14.5)	0.436^a^
Hypertension, *n* (%)	244 (53.9)	2704 (44.1)	<0.001^a^
Diabetes, *n* (%)	66 (14.6)	548 (8.9)	<0.001a

a Chi-square test.

b *t* test.

**Table 2.** Baseline characteristics of the WC analytical sample (*n* = 5538) by dementia status

	Dementia	No dementia	*P*‐Value
	(*n* = 432)	(*n* = 5106)	
WC (women/men, cm), *n* (%)			0.221^a^
Normal (≤88/102)	205 (47.5)	2586 (50.6)	
High/abdominal obesity (>88/102)	227 (52.5)	2520 (49.4)	
Age (years), mean (SD)	72.2 (7.9)	62.7 (9.0)	<0.001^b^
BMI + WC (6 categories)			0.547^a^
Normal BMI + WC	99 (23.9)	1261 (25.5)	
Normal BMI + High WC	8 (1.9)	75 (1.5)	
Obese + normal WC	94 (22.7)	1209 (24.5)	
Obese + high WC	214 (51.6)	2393 (48.5)	
Men, *n* (%)	192 (44.4)	2323 (45.5)	0.711^a^
APOE ε4 carrier, *n* (%)	169 (39.1)	1212 (23.7)	<0.001^a^
Education, *n* (%)			<0.001^a^
No qualifications	212 (49.1)	1755 (34.4)	
A-level	148 (34.3)	1984 (38.9)	
Higher	72 (16.7)	1367 (26.8)	
Unmarried, *n* (%)	196 (45.4)	1562 (30.6)	<0.001^a^
No physical activity, *n* (%)	29 (6.7)	189 (3.7)	0.003^a^
Current smokers, *n* (%)	55 (12.7)	725 (14.2)	0.441^a^
Hypertension, *n* (%)	239 (55.3)	2438 (47.7)	0.003^a^
Diabetes, *n* (%)	63 (14.6)	503 (9.9)	0.002^a^

a Chi-square test.

b *t* test.

### Associations between baseline BMI and dementia risk during follow-up

**Figure 2.**
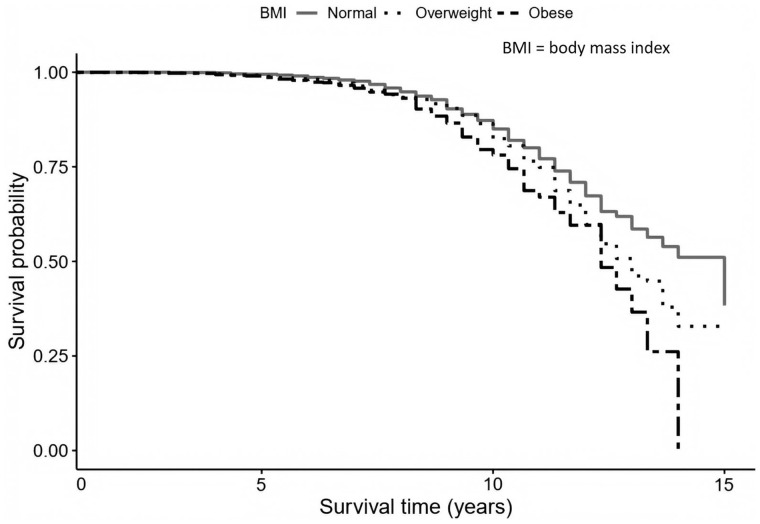
Survival plot representing the risk of dementia by body-weight groups.

The results from the Cox proportional hazard regressions investigating the relationships between baseline BMI and dementia onset during the average period of 11-year follow-up are presented in Table 3. The Cox proportional hazard regression in model 1 showed that participants with a BMI ≥ 30 kg/m^2^ at baseline had a 35% greater risk of dementia (HR = 1.35; 95% CI, 1.09–1.61; *P* < 0.05) than participants with normal BMI. Further adjustments for education and marital status (Model 2) did not substantially change this association (HR = 1.31; 95% CI, 1.04–1.58; *P* < 0.05). Obesity remained associated with a higher risk of dementia at follow-up even after adjusting for hypertension and smoking status (Model 3, HR = 1.34; 95% CI, 1.07–1.61; *P* < 0.05). In the fully adjusted model, additionally controlled for diabetes and hypertension, the association between obesity and dementia was slightly decreased (Model 4, HR = 1.31, 95% CI, 1.03–1.59; *P* < 0.05). There was no evidence of interactions between BMI and APOE (*P* = 0.141), sex (*P* = 0.689), hypertension (*P* = 0.823) or diabetes (*P* = 0.941). By comparing the AIC values for each model, it was confirmed that the best model fit with the lowest AIC was the fully adjusted model. Figure 2 presents the cumulative hazard estimates for BMI levels (normal, overweight and obese) in relation to dementia incidence by survival age.

**Figure 3.**
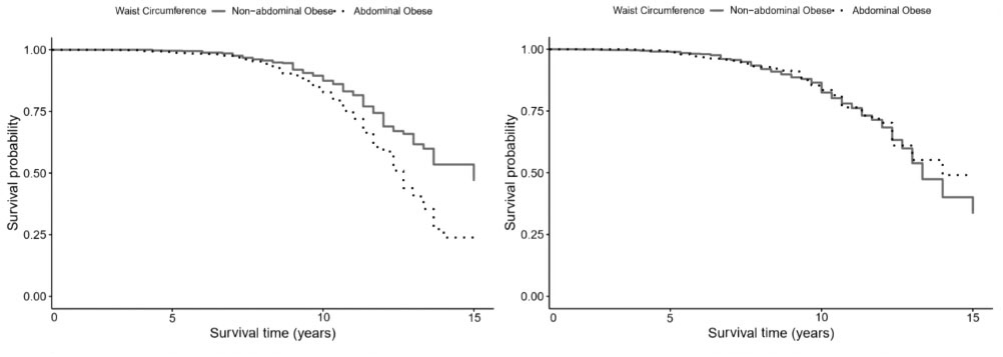
Survival plot representing the risk of dementia by waist circumference levels, in women (left panel) and men (right panel).

**Table 3.** Cox proportional hazards models of BMI at baseline and subsequent dementia

	Hazard ratios (95% confidence interval)			
	Model 1^a^	Model 2	Model 3	Model 4
BMI				
Normal (Ref^b^)	1	1	1	1
Overweight	1.24 (1.00, 1.48)	1.25 (1.01, 1.49)	1.29 (1.05, 1.53)*	1.27 (1.03, 1.51)*
Obese	1.35 (1.09, 1.61)*	1.31 (1.04, 1.58)*	1.34 (1.07, 1.61)*	1.31 (1.03, 1.59)*
Age (Continuous)	1.14 (1.13, 1.15)***	1.14 (1.13, 1.15)***	1.14 (1.13, 1.15)***	1.14 (1.13, 1.15)***
Sex				
Men (Ref)	1	1	1	1
Women	0.86 (0.66, 1.06)	0.78 (0.57, 0.99)*	0.79 (0.58, 1.00)*	0.80 (0.59, 1.01)*
APOE ɛ4 carrier				
No (Ref)	1	1	1	1
Yes	2.33 (2.13, 2.53)***	2.35 (2.15, 2.55)***	2.34 (2.14, 2.54)***	2.33 (2.13, 2.53)***
Education				
No qualifications (Ref)		1	1	1
A-level		0.87 (0.65, 1.09)	0.91 (0.69, 1.13)	0.89 (0.67, 1.11)
Degree		0.63 (0.34, 0.92)**	0.67 (0.38, 0.96)**	0.67 (0.38, 0.96)**
*Marital status				
Married (Ref)		1	1	1
Unmarried		1.09 (0.87, 1.31)	1.06 (0.84, 1.28)	1.06 (0.84, 1.28)
Physical activity				
Yes (Ref)			1	1
No			1.38 (0.99, 1.77)	1.34 (0.95, 1.73)
Smoker status				
Non-smoker (Ref)			1	1
Current smoker			1.73 (1.43, 2.03)***	1.70 (1.40, 2.00)***
Hypertension diagnosed				
No (Ref)				1
Yes				0.91 (0.71, 1.11)
Diabetes diagnosed				
No (Ref)				1
Yes				1.38 (1.10, 1.66)*
Observations	6582	6582	6582	6582

* *P*< 0.05;

** *P* < 0.01;

*** *P* < 0.001.

a Model 1 adjusted for age, sex and APOE ɛ4 at baseline. Model 2 based on Model 1 further adjusted for education and marital status at baseline. Model 3 based on Model 2 further adjusted for smoking status and physical activity at baseline. Model 4 based on Model 3 further adjusted for hypertension and diabetes at baseline.

b Ref, reference group.

### Associations between baseline WC and dementia risk

Table 4 (women, *n* = 3023) and Table 5 (men, *n* = 2515) present the sex-stratified results from the Cox proportional hazard regressions that estimated the association between baseline levels of WC and dementia incidence. The fully adjusted model in Table 4 indicates that women with abdominal obesity at baseline had a 39% increased risk of dementia (HR = 1.39; 95% CI, 1.12–1.66; *P* < 0.05) compared with those with non-abdominal obesity. However, no association with dementia was observed among men (HR = 0.84; 95% CI, 0.55–1.19; *P* = 0.23). Figure 3 depicts the survival plots for dementia risk by WC separately for women and men.

**Table 4.** Cox proportional hazards models of women WC at baseline and subsequent dementia

	Hazard ratios (95% confidence interval)			
	Model 1	Model 2	Model 3	Model 4
WC				
Normal (Ref^b^)	1	1	1	1
Abdominal obesity	1.46 (1.20, 1.72)**	1.45 (1.19, 1.71)**	1.45 (1.19, 1.71)**	1.39 (1.12, 1.66)*
Age (continuous)	1.15 (1.14, 1.16)***	1.15 (1.13, 1.17)***	1.15 (1.13, 1.17)***	1.15 (1.13, 1.17)***
APOE ɛ4 carrier				
No (Ref)	1	1	1	1
Yes	2.41 (2.15, 2.67)***	2.43 (2.17, 2.69)***	2.42 (2.16, 2.68)***	2.40 (2.14, 2.66)***
Education				
No qualifications (Ref)		1	1	1
A-level		0.90 (0.60, 1.20)	0.92 (0.61, 1.23)	0.92 (0.61, 1.23)
Degree		1.17 (0.80, 1.54)	1.22 (0.85, 1.59)	1.26 (0.88, 1.64)
Marital status				
Married (Ref)		1	1	1
Unmarried		1.11 (0.83, 1.39)	1.08 (0.80, 1.36)	1.08 (0.80, 1.36)
Physical activity				
Yes (Ref)			1	1
No			1.07 (0.60, 1.54)	1.07 (0.60, 1.54)
Smoker status				
Non-smoker (Ref)			1	1
Current smoker			1.87 (1.48, 2.26)**	1.84 (1.45, 2.23)**
Hypertension diagnosed				
No (Ref)				1
Yes				1.03 (0.76, 1.30)
Diabetes diagnosed				
No (Ref)				1
Yes				1.41 (0.97, 2.05)
Observations	3023	3023	3023	3023

* *P* < 0.05;

** *P* < 0.01;

*** *P* < 0.001.

a Model 1 adjusted for age and APOE ɛ4 at baseline. Model 2 based on Model 1 further adjusted for education and marital status at baseline. Model 3 based on Model 2 further adjusted for smoking status and physical activity at baseline. Model 4 based on Model 3 further adjusted for hypertension and diabetes at baseline.

b Ref, reference group.

**Table 5.** Cox proportional hazards models of men WC at baseline and subsequent dementia

	Hazard ratios (95% confidence interval)			
	Model 1^a^	Model 2	Model 3	Model 4
WC				
Normal (Ref^b^)	1	1	1	1
Abdominal obesity	0.86 (0.57, 1.15)	0.84 (0.55, 1.13)	0.85 (0.56, 1.14)	0.84 (0.55, 1.13)
Age (continuous)	1.13 (1.11, 1.15)***	1.12 (1.10, 1.14)***	1.13 (1.11, 1.15)***	1.13 (1.11, 1.15)***
APOE ɛ4 carrier				
No (Ref)	1	1	1	1
Yes	2.19 (1.89, 2.49)***	2.24 (1.94, 2.54)***	2.22 (1.92, 2.52)***	2.21 (1.91, 2.51)***
Education				
No qualification (Ref)		1	1	1
A-level		0.83 (0.51, 1.15)	0.87 (0.55, 1.19)	0.84 (0.52, 1.16)
Degree		0.40 (0.26, 0.61)***	0.42 (0.28, 0.63)***	0.41 (0.27, 0.62)***
Marital status				
Married (Ref)		1	1	1
Unmarried		1.06 (0.73, 1.39)	1.04 (0.71, 1.37)	1.02 (0.69, 1.35)
Physical activity				
Yes (Ref)			1	1
No			1.11 (0.42, 1.80)	1.02 (0.33, 1.70)
Smoker status				
Non-smoker (Ref)			1	1
Current smoker			1.56 (1.12, 2.00)	1.55 (1.11, 1.99)
Hypertension diagnosed				
No (Ref)				1
Yes				0.87 (0.58, 1.16)
Diabetes diagnosed				
No (Ref)				1
Yes				1.48 (1.08, 1.88)
Observations	2515	2515	2515	2515

* *P* < 0.05;

** *P* < 0.01;

*** *P* < 0.001.

a Model 1 Adjusted for age and APOE ɛ4 at baseline. Model 2 based on Model 1 further adjusted for education and marital status at baseline. Model 3 based on Model 2 further adjusted for smoking status and physical activity at baseline. Model 4 based on Model 3 further adjusted for hypertension and diabetes at baseline.

b Ref, reference group.

### Associations between a combination of baseline BMI + WC levels and dementia risk

When compared with normal BMI + WC levels, being obese and having high WC was associated with a 1.28-fold increase in dementia risk (95% CI, 1.03–1.53; *P* < 0.05) (Table 6). There is no evidence showing the association between groups of normal BMI + high WC (HR = 1.01; 95% CI, 0.26–1.76; *P* = 0.97), obese + normal WC (HR = 1.32; 95% CI, 1.03–1.61; *P* = 0.06) and dementia.

**Table 6.** Cox proportional hazards models of BMI and WC measures at baseline and subsequent dementia

	Hazard ratios (95% confidence interval)			
	Model 1^a^	Model 2	Model 3	Model 4
Normal BMI + WC (Ref)	1	1	1	1
Normal BMI + high WC	1.07 (0.33, 1.81)	1.09 (0.35, 1.83)	1.01 (0.26, 1.76)	1.01 (0.26, 1.76)
Obese+ normal WC	1.28 (0.99, 1.57)	1.30 (1.01, 1.59)	1.33 (1.04, 1.62)	1.32 (1.03, 1.61)
Obese+ high WC	1.31 (1.07, 1.55)*	1.30 (1.06, 1.54)*	1.31 (1.07, 1.55)*	1.28 (1.03, 1.53)*
Age (continuous)	1.14 (1.13, 1.15)***	1.14 (1.13, 1.15)***	1.14 (1.13, 1.15)***	1.14 (1.13, 1.15)***
Sex				
Men (Ref)	1	1	1	1
Women	0.86 (0.66, 1.06)	0.79 (0.58, 1.00)*	0.79 (0.58, 1.00)*	0.81 (0.60, 1.02)*
APOE ɛ4 carrier				
No (Ref)	1	1	1	1
Yes	2.34 (2.14, 2.54)***	2.36 (2.16, 2.56)***	2.35 (2.15, 2.55)***	2.34 (2.14, 2.54)***
Education				
No qualifications (Ref)		1	1	1
A-level		0.88 (0.66, 1.10)	0.92 (0.69, 1.15)	0.90 (0.67, 1.13)
Degree		0.64 (0.35, 0.93)**	0.67 (0.38, 0.96)**	0.67 (0.38, 0.96)**
Marital status				
Married (Ref)		1	1	1
Unmarried		1.09 (0.87, 1.31)	1.06 (0.84, 1.28)	1.05 (0.83, 1.27)
Physical activity				
Yes (Ref)			1	1
No			1.41 (1.02, 1.99)	1.37 (0.98, 1.76)
Smokers status				
Non-smokers (Ref)			1	1
Current smokers			1.68 (1.37, 1.99)***	1.65 (1.34, 1.96)**
Hypertension diagnosed				
No (Ref)				1
Yes				0.92 (0.72, 1.12)
Diabetes diagnosed				
No (Ref)				1
Yes				1.41 (1.13, 1.69)*
Observations	5353	5353	5353	5353

* *P* < 0.05;

** *P* < 0.01;

*** *P* < 0.001.

a Model 1 Adjusted for age, sex and APOE ɛ4 at baseline. Model 2 based on Model 1 further adjusted for education and marital status at baseline. Model 3 based on Model 2 further adjusted for smoking status and physical activity at baseline. Model 4 based on Model 3 further adjusted for hypertension and diabetes at baseline.

b Ref, reference group.

### Sensitivity analyses

The first sensitivity analysis explored the inclusion of the underweight category in the analytical sample and showed that obesity remained associated with a higher risk of developing dementia compared with participants with a normal weight, independent of all selected covariates (HR = 1.32; 95% CI, 1.04–1.60; *P* < 0.05) (Supplementary Table S1, available as Supplementary data at *IJE* online**)**. However, we did not find an association between underweight and subsequent dementia onset, perhaps due to the lower number of participants in this group. The final two sensitivity analyses have investigated a 3-level classification of BMI (normal, overweight and obese) within an imputed dataset of *n* = 19 184 (of whom *n* = 1199, 6.3%, were those who developed dementia during the average 11-year follow-up). The results presented in Supplementary Table S2, available as Supplementary data at *IJE* online, are consistent with those from the main analyses, indicating that being obese was associated with an increased probability of dementia independence (HR = 1.21; 95% CI, 1.01–1.41; *P* < 0.05) independent of all selected covariates including hypertension, diabetes and lifestyle behaviours. Supplementary Table S3, available as Supplementary data at *IJE* online, shows that in individuals with abdominal obesity, the risk of developing dementia was 41% greater than in those with normal WC (HR = 1.41; 95% CI, 1.29–1.53; *P* < 0.001).

## Discussion

This study examined the risk of dementia over an up to 15-year follow-up period in relation to baseline body weight status in a nationally representative sample of the older population in England. The main findings are 2-fold. Firstly, our results provide further evidence of a positive and independent association between obesity and dementia risk, after controlling for potential confounders, including APOE-ε4 carrier status. Secondly, our findings show that larger abdominal obesity was also indicative of increased dementia incidence, particularly in women. This is consistent with previous reports showing that high BMI, as well as high WC, contribute to the development of late-life dementia.^28^ ^40–44^ Nonetheless, there is some evidence that contradicts this conclusion.^45^ ^46^ The same pattern of dementia incidence was found for both men and women, which differs to a certain extent from the previous findings showing that women with higher BMI (obesity-level) proved to have a higher risk of dementia.^25^ The present study also provides further support that a combination of high BMI and WC was associated with higher odds of dementia incidence.

The potential mechanisms by which adiposity contributed to the risk of dementia involved comorbidities, genetics and inflammatory processes. Although some evidence suggests that this association could potentially be mediated by other comorbidities, such as hypertension, cardiovascular diseases and diabetes,^11^ ^47^ we did not find evidence to support these notions. Although APOE-ε4 is a strong genetic risk factor for dementia,^48^ we found no evidence that APOE-ε4 status has an interaction effect with obesity in this analysis, which is consistent with results from the Finnish CAIDE study.^49^ The precise biological mechanisms of how APOE-ε4 modifies the effects of obesity on the risk of late-life dementia are still poorly understood. It has been suggested that APOE-ε4 triggers inflammatory cascades that lead to neurovascular dysfunction, i.e. blood–brain barrier failure, exposure of toxic derivational proteins from the blood into the brain and decrease of small vessels’ length.^48^ Several endocrine axes bridging the brain and periphery were also suggested to influence hippocampal and hypothalamic functions by involving adipokines and adipose tissue.^50^

Cumulatively, the results reported in the present study may present important implications for the prevention and delay of dementia onset. Indeed, obesity is suggested as a risk factor for dementia, and its prevalence has experienced a rapid increase, ∼5-fold, over the past 40 years. The ongoing obesity epidemic is predicted to cause a surge of dementia incidences in the near future. The results reported in the present study highlight the need to consider potential public health interventions that will address obesity and prevent dementia. However, only a few countries have formulated national dementia plans at present^51^ and most interventions to date focus on lifestyle factors other than obesity. Further research providing more evidence is needed.

### Methodological considerations

Strength of the present study represents the inclusion of a national middle-aged representative sample of the English population, with a relatively equal distribution of both men and women, improving the generalizability of the findings. Moreover, the regular nurse visits and the use of standardized and objective BMI measurements ensured the quality of data employed in our analyses. Besides, dementia cases were diagnosed by physicians, and we benefited from repeated measures of dementia reports that enabled us to capture cumulative events over time and allowed us to conduct a survival analysis. To date, this is the first study to examine the association between middle-aged and older ELSA participants with obesity and subsequent related dementia risk in a large representative sample of English adults, which might have potential implications for interventions designed to intervene in terms of modifiable risk factors for preventing dementia or delaying its onset.

Nonetheless, this study has several potential limitations. One notable drawback is related to the length of the follow-up, which may be questionable when monitoring dementia development, and especially when trying to distinguish the potential risk factors from early symptomatology occurring during the prodromal stage.^52^ In addition, there were fewer diagnosed cases with dementia in this analytical sample than expected in general populations when comparing the estimates of dementia prevalence in those >65 years old in the UK. The ascertainment of a dementia diagnosis is still challenging, therefore it is feasible that the presented dementia cases may be underestimated. Another limitation of this research is that the identification of dementia is based on physician diagnosis. Because of the small numbers with dementia, this study focused on all-types of dementia and it was inappropriate to conduct further stratification analyses by subcategories of dementia such as Alzheimer’s disease and vascular dementia.

Similarly, this study was unable to investigate BMI at an underweight level, given that only a reduced number of participants who were underweight at their baseline were subsequently diagnosed with dementia; the proportion categorized as underweight was much lower than expected. Thus, there was an insufficient number of cases to examine the risk of dementia in individuals who were underweight at baseline. Additional research with larger samples is required to understand these mechanisms and further studies should consider earlier measurements of obesity, as people tend to lose weight at the time of dementia diagnosis.^53^ Lastly, given the observational nature of this study, although longitudinal, we cannot infer causality or eliminate the role of residual confounding.

## Conclusion

The findings of this study showed that being overweight or obese was associated with subsequent higher rates of dementia incidence. Furthermore, we found a higher dementia incidence in women with abdominal obesity. Given the ongoing increase in obesity levels around the world, these findings have important implications in terms of designing appropriate interventions for preventing and managing contributing factors to obesity and associated consequences, including dementia onset. Identifying individuals at higher risk of dementia may play a role in future treatments and interventions to reduce their future risk of dementia.

## Funding

The work was supported by the National Institute on Aging (grants 5218182, R01AG7644-01A1, and R01AG017644). The English Longitudinal Study of Ageing is funded by the National Institute on Aging (grant R01AG7644) and by a consortium of UK government departments coordinated by the Economic and Social Research Council (ESRC) and the Office for National Statistics. O.A. is jointly funded by the National Institute on Aging and the National Institute for Health Research (NIHR) (NIHR Post-Doctoral Fellowship - PDF-2018–11-ST2-020). The funders had no role in the design and conduct of the study; collection, management, analysis, and interpretation of the data; preparation, review, or approval of the manuscript; and decision to submit the manuscript for publication. The English Longitudinal Study of Ageing (ELSA) was developed by a team of researchers based at University College London, the Institute for Fiscal Studies and the National Centre for Social Research.

## Conflict of interest

None declared.

## Supplementary Material

dyaa099_Supplemetary_Data
