# Supplementary material for: Higher risk of dementia in English older individuals who are overweight or obese
Source: Int J Epidemiol. 2020 Jun 23;49(4):1353–65. doi: 10.1093/ije/dyaa099 (PMC7660153; doi:10.1093/ije/dyaa099)
Supplement: dyaa099_Supplemetary_Data [file dyaa099_supplemetary_data.docx]

| **Supplementary material**  **Supplementary tables** | | | | |  |
| --- | --- | --- | --- | --- | --- |
| **Table S1. Multivariate analysis for mid-life BMI with four groups (underweight, overweight, obese compared to normal BMI) and subsequent dementia** | | | | |  |
|  | | | | |  |
|  | Hazard Ratios (95% Confidence Interval) | | | |  |
|  |  | | | |  |
|  | Model 1 | Model 2 | Model 3 | Model 4 | |
|  | | | | | |
| BMI |  |  |  |  | |
| Normal (Ref) | 1 | 1 | 1 | 1 | |
| Underweight | 1.64 (0.55, 2.73) | 1.71 (0.62, 2.80) | 1.62 (0.53, 2.71) | 1.66 (0.57, 2.75) | |
| Overweight | 1.25 (1.01, 1.49) | 1.27 (1.03, 1.51)^*^ | 1.30 (1.06, 1.54)^*^ | 1.29 (1.05, 1.53)^*^ | |
| Obese | 1.37 (1.10, 1.64)^*^ | 1.33 (1.06, 1.60)^*^ | 1.36 (1.09, 1.63)^*^ | 1.32 (1.04, 1.60)^*^ | |
| Age (continuous) | 1.14 (1.13, 1.15)^***^ | 1.14 (1.13, 1.15)^***^ | 1.14 (1.13, 1.15)^***^ | 1.14 (1.13, 1.15)^***^ | |
| Sex |  |  |  |  | |
| Men (Ref) | 1 | 1 | 1 | 1 | |
| Women | 0.85 (0.65, 1.05) | 0.78 (0.57, 0.99)^*^ | 0.78 (0.57, 0.99)^*^ | 0.80 (0.59, 1.01)^*^ | |
| APOE e4 carrier |  |  |  |  | |
| No (Ref) | 1 | 1 | 1 | 1 | |
| Yes | 2.34 (2.14, 2.54)^***^ | 2.36 (2.16, 2.56)^***^ | 2.35 (2.15, 2.55)^***^ | 2.34 (2.14, 2.54)^***^ | |
| Education |  |  |  |  | |
| No Qualification (Ref) |  | 1 | 1 | 1 | |
| A level |  | 0.86 (0.64, 1.08) | 0.90 (0.68, 1.12) | 0.89 (0.67, 1.11) | |
| Degree |  | 0.63 (0.34, 0.92)^**^ | 0.67 (0.38, 0.96)^*^ | 0.67 (0.38, 0.96)^*^ | |
| Marital Status |  |  |  |  | |
| Married (Ref) |  | 1 | 1 | 1 | |
| Unmarried |  | 1.09 (0.87, 1.31) | 1.05 (0.83, 1.27) | 1.05 (0.83, 1.27) | |
| Physical Activity |  |  |  |  | |
| No (Ref) |  |  | 1 | 1 | |
| Yes |  |  | 1.37 (0.98, 1.76) | 1.34 (0.95, 1.73) | |
| Smokers Status |  |  |  |  | |
| Non-smokers (Ref) |  |  | 1 | 1 | |
| Current Smokers |  |  | 1.73 (1.43, 2.03)^***^ | 1.70 (1.40, 2.00)^***^ | |
| Hypertension Diagnosed |  |  |  |  | |
| No (Ref) |  |  |  | 1 | |
| Yes |  |  |  | 0.91 (0.71, 1.11) | |
| Diabetes Diagnosed |  |  |  |  | |
| No (Ref) |  |  |  | 1 | |
| Yes |  |  |  | 1.38 (1.10, 1.66)^*^ | |
|  | | | | | |
| Observations | 6,582 | 6,582 | 6,582 | 6,582 | |
| Note: | *p<0.05; **p<0.01; ***p<0.001 | | | |  |

BMI: body mass index; Ref: reference group

Model 1 Adjusted for age, sex and APOE e4 at baseline

Model 2 based on Model 1 further adjusted for education and marital status at baseline

Model 3 based on Model 2 further adjusted for smoking status and physical activity at baseline

Model 4 based on Model 3 further adjusted for hypertension and diabetes at baseline

| **Table S2. Cox proportional hazards models of BMI at mid-life and risk of dementia in an imputed dataset** | | | | | |
| --- | --- | --- | --- | --- | --- |
|  | | | | | |
|  | | Hazard Ratios (95% Confidence Interval) | | | |
|  | |  | | | |
|  | | Model 1 | Model 2 | Model 3 | Model 4 |
|  | | | | | |
| BMI | |  |  |  |  |
| Normal (Ref) | | 1 | 1 | 1 | 1 |
| Overweight | | 1.65 (1.49, 1.81)^***^ | 1.63 (1.47, 1.79)^***^ | 1.62 (1.46, 1.78)^***^ | 1.62 (1.46, 1.78)^***^ |
| Obesity | | 1.35 (1.16, 1.54)^**^ | 1.25(1.06, 1.44)^*^ | 1.23 (1.04, 1.42)^*^ | 1.21 (1.01, 1.41)^*^ |
| Age (Continuous) | | 1.13 (1.12, 1.14)^***^ | 1.12 (1.11, 1.13)^***^ | 1.11 (1.10, 1.12)^***^ | 1.11 (1.10, 1.12)^***^ |
| Sex | |  |  |  |  |
| Men (Ref) | | 1 | 1 | 1 | 1 |
| Women | | 1.32 (1.21, 1.43)^***^ | 1.45 (1.33, 1.57)^***^ | 1.48 (1.36, 1.60)^***^ | 1.47 (1.35, 1.59)^***^ |
| Education | |  |  |  |  |
| No Qualification (Ref) | |  | 1 | 1 | 1 |
| A level | |  | 0.65 (0.51, 0.75)^***^ | 0.70 (0.56, 0.84)^***^ | 0.70 (0.56, 0.84)^***^ |
| Degree | |  | 0.49 (0.31, 0.67)^***^ | 0.54 (0.36, 0.72)^***^ | 0.54 (0.36, 0.72)^***^ |
| Marital Status | |  |  |  |  |
| Married (Ref) | |  | 1 | 1 | 1 |
| Unmarried | |  | 1.00 (0.87, 1.13) | 0.93 (0.80, 1.06) | 0.93 (0.80, 1.06) |
| Physical Activity | |  |  |  |  |
| Yes (Ref) | |  |  | 1 | 1 |
| No | |  |  | 2.35 (2.19, 2.51)^***^ | 2.32 (2.16, 2.48)^***^ |
| Smokers Status | |  |  |  |  |
| Non-smokers (Ref) | |  |  | 1 | 1 |
| Current Smokers | |  |  | 1.54 (1.36, 1.72)^***^ | 1.54 (1.36, 1.72)^***^ |
| Hypertension Diagnosed | |  |  |  |  |
| No (Ref) | |  |  |  | 1 |
| Yes | |  |  |  | 1.00 (0.88, 1.12) |
| Diabetes Diagnosed | |  |  |  |  |
| No (Ref) | |  |  |  | 1 |
| Yes | |  |  |  | 1.10 (0.93, 1.27) |
|  | | | | | |
| Observations | | 19,184 | 19,184 | 19,184 | 19,184 |
|  | | | | | |
| Note: | | *p<0.05; **p<0.01; ***p<0.001 | | | |
|  | BMI: body mass index, Ref: reference group | | | | |
|  | Model 1 Adjusted for age and sex at baseline | | | | |
|  | Model 2 based on Model 1 further adjusted for education and marital status at baseline | | | | |
|  | Model 3 based on Model 2 further adjusted for smoking status and physical activity at baseline | | | | |
|  | Model 4 based on Model 3 further adjusted for hypertension, diabetes and at baseline | | | | |

| **Table S3. Cox proportional hazards models of WC at baseline and risk of dementia in an imputed dataset** | | | | | |
| --- | --- | --- | --- | --- | --- |
|  | | | | | |
|  | | Hazard Ratios (95% Confidence Interval) | | | |
|  | |  | | | |
|  | | Model 1 | Model 2 | Model 3 | Model 4 |
|  | | | | | |
| WC | |  |  |  |  |
| Normal (Ref) | | 1 | 1 | 1 | 1 |
| Abdominal obesity | | 1.41 (1.29, 1.53)^***^ | 1.38 (1.26, 1.50)^***^ | 1.40 (1.28, 1.52)^***^ | 1.41 (1.29, 1.53)^***^ |
| Age (Continuous) | | 1.13 (1.12, 1.14)^***^ | 1.13 (1.12, 1.14)^***^ | 1.12 (1.11, 1.13)^***^ | 1.12 (1.11, 1.13)^***^ |
| Sex | |  |  |  |  |
| Men (Ref) | | 1 | 1 | 1 | 1 |
| Women | | 0.72(0.62, 0.83)^***^ | 0.68 (0.56, 0.80)^***^ | 0.67 (0.55, 0.79)^***^ | 0.67 (0.55, 0.79)^***^ |
| Education | |  |  |  |  |
| No Qualification (Ref) | |  | 1 | 1 | 1 |
| A level | |  | 0.75 (0.62, 0.88)^***^ | 0.81 (0.67, 0.95)^***^ | 0.81 (0.67, 0.95)^***^ |
| Degree | |  | 0.62 (0.45, 0.79)^***^ | 0.68 (0.51, 0.85)^***^ | 0.68 (0.51, 0.85)^***^ |
| Marital Status | |  |  |  |  |
| Married (Ref) | |  | 1 | 1 | 1 |
| Unmarried | |  | 0.98(0.85, 1.11) | 0.93 (0.80, 1.06) | 0.94 (0.81, 1.07) |
| Physical Activity | |  |  |  |  |
| Yes (Ref) | |  |  | 1 | 1 |
| No | |  |  | 2.56 (2.40, 2.72)^***^ | 2.55 (2.39, 2.71)^***^ |
| Smokers Status | |  |  |  |  |
| Non-smokers (Ref) | |  |  | 1 | 1 |
| Current Smokers | |  |  | 1.44 (1.26, 1.62)^***^ | 1.44 (1.26, 1.62)^***^ |
| Hypertension Diagnosed | |  |  |  |  |
| No (Ref) | |  |  |  | 1 |
| Yes | |  |  |  | 0.95 (0.83, 1.07) |
| Diabetes Diagnosed | |  |  |  |  |
| No (Ref) | |  |  |  | 1 |
| Yes | |  |  |  | 1.01 (0.84, 1.18) |
|  | | | | | |
| Observations | | 19,184 | 19,184 | 19,184 | 19,184 |
|  | | | | | |
| Note: | | *p<0.05; **p<0.01; ***p<0.001 | | | |
|  | WC: waist circumference, Ref: reference group | | | | |
|  | Model 1 Adjusted for age and sex at baseline | | | | |
|  | Model 2 based on Model 1 further adjusted for education and marital status at baseline | | | | |
|  | Model 3 based on Model 2 further adjusted for smoking status and physical activity at baseline | | | | |
|  | Model 4 based on Model 3 further adjusted for hypertension, diabetes and at baseline | | | | |
